# Lung abscess due to *Streptococcus intermedius* associated with SARS CoV‐2 infection in pregnancy: Unusual presentation and complication of a commensal bacteria during pregnancy

**DOI:** 10.1002/ccr3.6763

**Published:** 2023-01-03

**Authors:** Cristian Orlando Porras Bueno, Sharon Julieth González Trillos, Diana Jimena Cano Rosales, Edgar Augusto Bernal García

**Affiliations:** ^1^ Faculty of Health Sciences Autonomous University of Bucaramanga Bucaramanga Colombia; ^2^ Department of Internal Medicine Fundación Oftalmológica de Santander Bucaramanga Colombia; ^3^ Research Department Instituto Neumológico del oriente Bucaramanga Colombia

**Keywords:** infectious diseases, lung abscess, pneumonia, bacterial, respiratory system, SARS‐CoV‐2

## Abstract

*Streptococcus intermedius* is a commensal bacterium reported in a few cases as the causative agent of brain and lung abscesses, pneumonia, and endocarditis. Lung abscesses due to *Streptococcus intermedius* are rare, especially in pregnancy. We describe the first case of lung abscess due to *Streptococcus intermedius* in a pregnant woman.

## Graphical abstract

*Streptococcus intermedius* is part of the commensal oral flora, but it has been described in a few cases as the causal agent of lung abscesses. We describe an unusual case of lung abscess caused by *Streptococcus intermedius* in pregnancy.

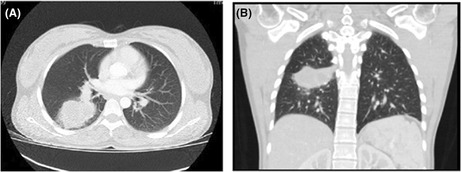

## 1 INTRODUCTION

*Streptococcus intermedius* is a catalase‐negative, gram‐positive cocci, whose most isolates are nonhemolytic with small colony‐forming species, which belongs to the *Streptococcus anginosus* group (SAG), which has also been referred to as the *Streptococcus milleri* group, which includes 3 organisms: *S. anginous*, *S. intermedius*, and *S. constellatus*.^1^ This bacterial group has not been recognized as a causative pathogen. However, with the presence of certain factors, SAG could induce noninvasive infections and also invasive infections after getting into sterile body sites, such as the blood and serosal cavity, which is why it could affect the tissues and organs of several systems of the body.^2^

The SAG species differ in the virulence factors that they produce. Because *S. intermedius* produces sialidase and hyaluronidase, which can destroy host tissues, converting them into nutrients for bacterial growth, whereas *S. constellatus* produces only hyaluronidase and *S. anginous* none of these.^3^ Probably, for this reason, *S. intermedius* has the ability to form abscesses in several body locations. However, its virulence factors are unclear at present.^4^ This feature gives it a unique distinction compared with other alpha‐hemolytic streptococcal species and makes its management require, in most cases, surgical intervention along with antibiotic therapy.^4^

*Streptococcus intermedius* is part of the commensal oral flora in humans, and it is frequently associated with brain and liver abscesses, but less frequently with pleuropulmonary infections including pneumonia, pleural effusion, and empyema, and in a few cases, it can also be the causal agent of lung abscesses.^1^, ^5^ Among the risk factors for these infections, smoking, alcoholism, dental diseases, chronic obstructive pulmonary disease, malignant neoplasms, liver cirrhosis, and diabetes have been described.^1^ Through this case report with a literature review, we discuss an acute presentation of *S. intermedius* lung abscess in a pregnant woman with radiological findings that make it difficult to distinguish it as a lung neoplasm, empyema, or abscess.

## 2 CASE REPORT

A 25‐year‐old woman with a 34‐week and 5‐day pregnancy, with a medical history of astigmatism, consulted the emergency department, stating that in the three previous weeks, she had presented with cough with hemoptysis, fatigue, headache, odynophagia, and 6 kg weight loss. On physical exam, vital signs were within the normal ranges, and a gravid uterus, fetal heart rate, and fetal movements were present. Initially, the severe acute respiratory syndrome coronavirus 2 (SARS‐CoV2) antigen test was negative. However, chest radiography showed a lung mass in the upper segment of the right lower lobe. Also, serial sputum smear microscopy for tuberculosis was negative.

**FIGURE 1:**
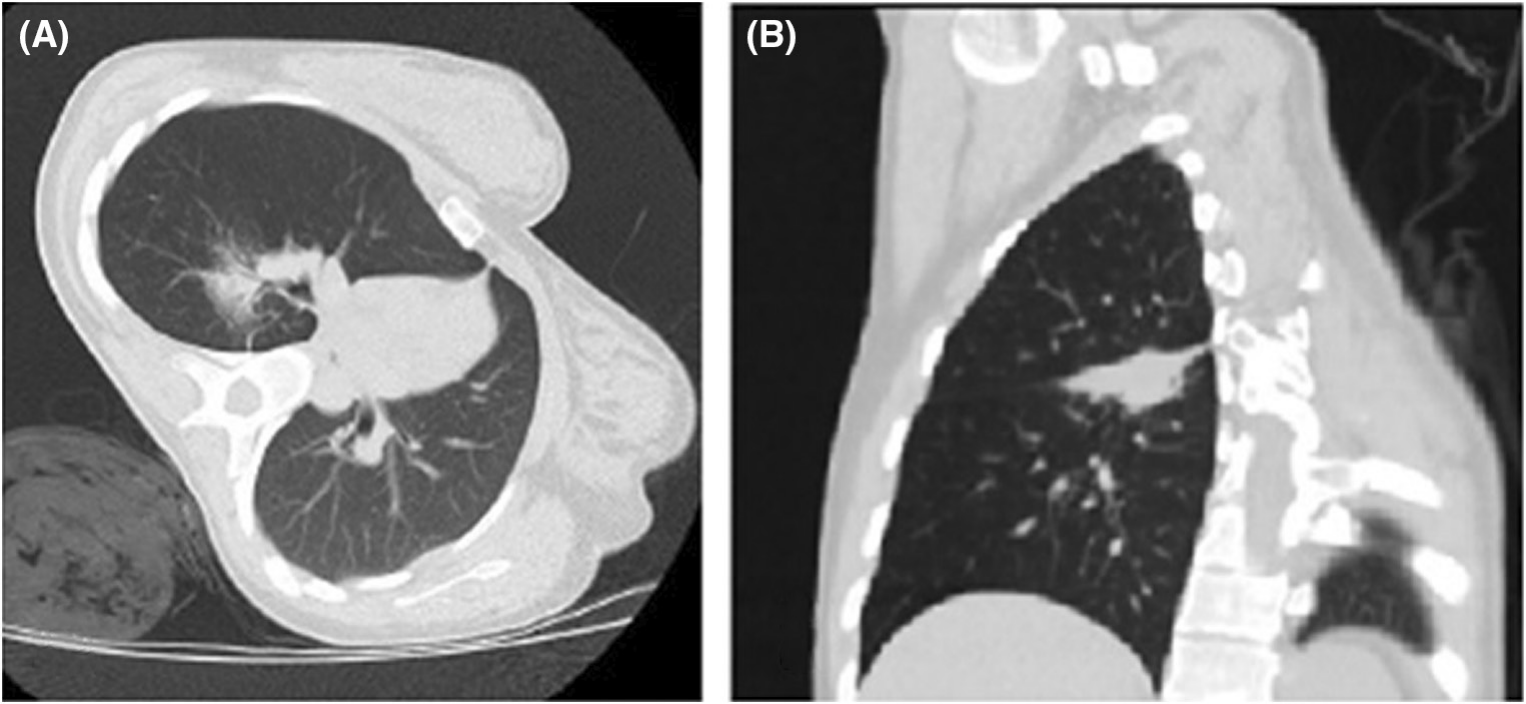
(A) Axial computed tomography view showing a lesion in the upper segment of the lower lobe on the right side. (B) Coronal computed tomography view.

Given the persistence of symptoms, a polymerase chain reaction (PCR) to SARS‐CoV2 was performed with a positive result, confirming a mild acute respiratory infection due to SARS‐CoV2 that evolved satisfactorily with management at home. Nevertheless, a high‐resolution chest computed tomography (CT) was taken, reporting a lung mass in the upper segment of the right lower lobe adjacent to the horizontal pulmonary fissure of origin to be determined (Figure 1). Then, a sputum PCR was performed for Mycobacterium tuberculosis and tuberculin test, which were negative, so given the characteristics of the mass, tuberculosis, or pulmonary mycosis were discarded. The Pneumology service performed a fibrobronchoscopy and bronchoalveolar lavage, obtaining negative cytology for malignancy and also a Gram stain, KOH test, smear microscopy for tuberculosis, and PCR for mycobacteria with negative results, so the patient was discharged with ambulatory management.

**FIGURE 2:**
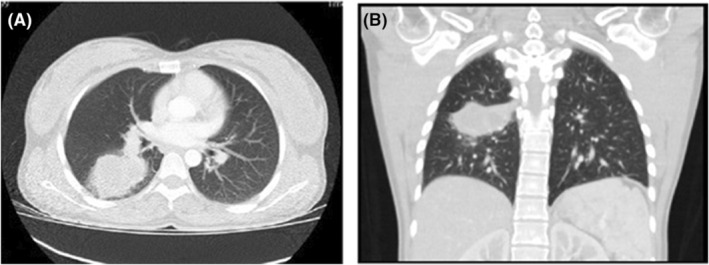
(A) Axial computed tomography view, shows an increase in the abscessed lesion in the upper segment of the lower lobe on the right side. (B) Coronal computed tomography view.

Ten days later, the patient was again consulted at the emergency department due to the persistence of respiratory symptoms. A high‐resolution chest CT reported an increase in the lung lesion size (Figure 2), so she was hospitalized. A lung biopsy was performed by interventional radiology after the pregnancy ended by cesarean section at 34 weeks and 5 days of the patient's pregnancy, draining purulent material. The newborn was female, with expected anthropometric measurements in relation to her prematurity and an Apgar score of 8 at 1 min and 10 at 10 min. The newborn did not present complications after birth; however, it required a kangaroo mother care intervention program.

**FIGURE 3:**
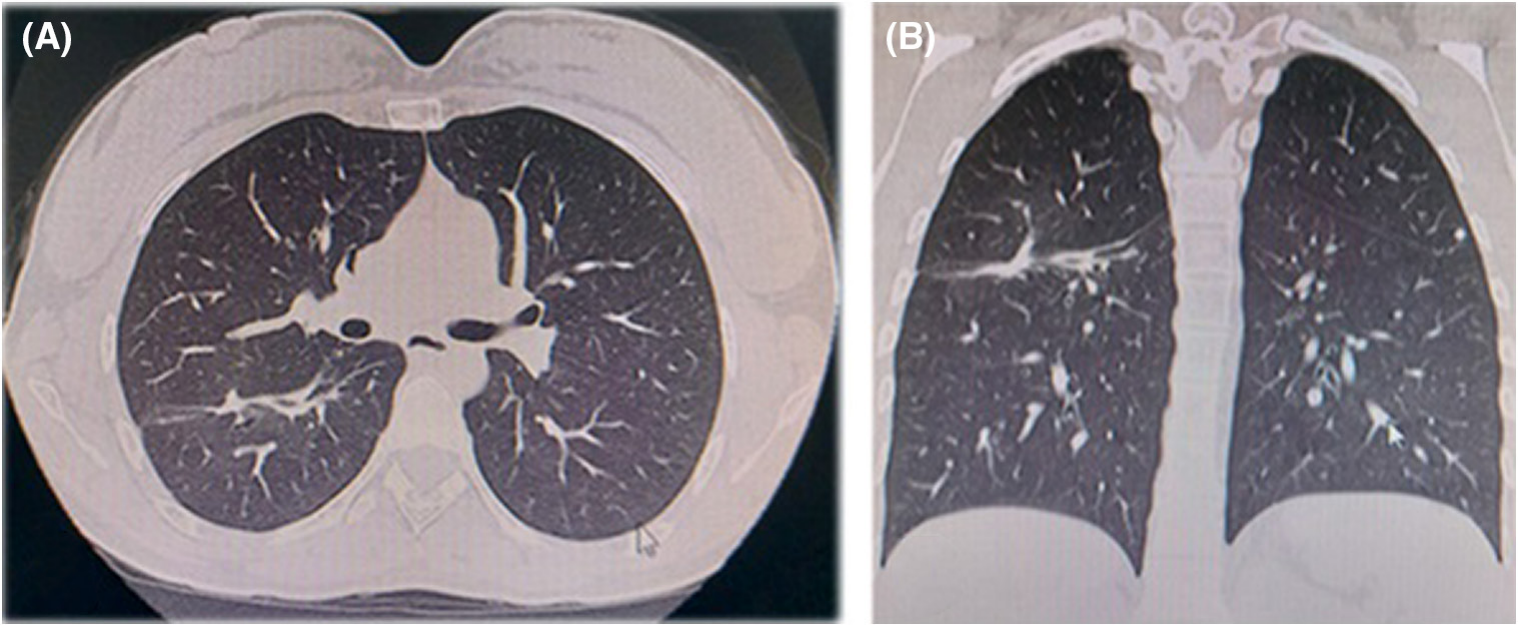
(A) Axial computed tomography view, showing thickening of the major fissure of the right lung associated with a small hyperdense image of irregular subpleural shape adjacent to the major fissure of the lower lobe, as residual changes in relation to pathological and surgical history. (B) Coronal computed tomography view.

The microscopical examination of the purulent drained material from the lung lesion reported an acute bronchopneumonic process. The KOH test, Chinese ink, and smear microscopy for tuberculosis studies were also performed with negative results. However, the culture was positive, isolating a *S. intermedius* with the usual antibiotic susceptibility profile. The antibiotic management in the hospital was started with ceftriaxone and, 4 days later, given a good response to treatment, the patient was discharged with amoxicillin for 1 month. After discharge, symptoms resolved 1 week later, and tomographic findings reversed progressively after 4 months (Figure 3).

## 3 DISCUSSION

*Streptococcus intermedius* infections have been described in a wide age range: For example, Nakagawa et al.^6^ reported a lung abscess and empyema caused by *S. intermedius* in an immunocompetent 6‐month‐old boy, whereas Yanagihara et al.^7^ reported a brain abscess as a complication of hepatopulmonary syndrome coexisting with interstitial pneumonia in a 76‐year‐old woman. However, the oldest patient reported was 80 years old and developed an empyema and a psoas abscess due to *S. intermedius*.^8^ Our patient was also in this wide age range. Regarding sex predominance, respiratory infections due to SAG have been described more frequently in male patients with comorbid diseases who are typically complicated by pleural effusion.^9^

Patients with infections due to *S. intermedius* usually present nonspecific symptoms such as fever, chills, and general discomfort among the cases described. However, additionally, patients would exhibit symptoms related to the body side affected by it, for example, seizures, headaches, nausea, vomiting in brain abscesses^10^, ^11^ or cough, sputum production, shortness of breath, hemoptysis in pneumonia, empyema, lung abscesses,^5^, ^12^, ^13^ which are uncommon clinical complications from *S. intermedius* with few cases currently described (Table 1). Moreover, chest pain, chest distress, and even respiratory failure have been described in pleural effusion and mediastinal abscess due to SAG, besides, odynophagia, and cervicodynia in oropharynx infections.^2^

**TABLE 1:** Articles related to lung abscess due to *Streptococcus intermedius*.

Age of publication—Article title	Type of article	Age	Gender	Medical history	Type of infection	Treatment	Outcome
2006—A case of pulmonary abscess in which Haemophilus parainfluenzae and *Streptococcus intermedius* were isolated by percutaneous needle aspiration	Case report	75 years old	Male	Esophago‐gastrectomy due to esophageal cancer	Lung abscess	Panipenem/Betamipron	Without data
2013—Pyogenic Brain and Lung Abscesses due to *Streptococcus intermedius*	Case report	36 years old	Male	Methamphetamine abuse	Lung and cerebral abscesses	Stereotaxic drainage of abscesses and unspecified intravenous antibiotic therapy	Without data
2015—The clinical features of respiratory infections caused by the *Streptococcus anginosus* group	Cross‐sectional study (30 patients −16 patients with *S. Intermedius*)	68.9 ± 14.2 years old (average)	73.3% (22) Male	Comorbidity diseases in 93.3% (28) Smoking history in 43.3% (13) Cerebrovascular disease in 40% (12) Neoplastic disease in 26.7% (8) Diabetes mellitus in 20% (6)	Pneumonia in 63.3% (19) Lung abscess in 16.7% (5) Bacterial pleurisy in 20% (6)	Antibiotic monotherapy in 83.3% (25) with Carbapenem 76% (19) Penicillin/beta‐lactamase inhibitors 12% (3) Macrolide 8% (2) Linezolid 4% (1) Drainage in 16.7% (5) Drainage + lung decortication in 46.7% (14)	In‐hospital mortality in 6.7% (2)
2016—*Streptococcus intermedius* Causing Necrotizing Pneumonia in an Immune Competent Female: A case report and literature Review	Case report	52 years old	Female	Asthma, former smoker	Lung abscess	Ceftriaxone for 14 days plus decortication and resection of right upper lung lobe and chest tube insertion	Recovery from his symptoms and tomographic findings
2017—Isolated *Streptococcus intermedius* pulmonary nodules	Case report	29 years old	Male	Congenital nistagmus and umbilical hernia	Lung abscess	Ceftriaxone for 4 weeks	Recovery from his symptoms and tomographic findings
2020—A case of an 80‐year‐old man with Empyema and Psoas Abscess	Case report	80 years old	Male	Ischemic stroke, spinal stenosis, gastrectomy, coronary artery disease	Right empyema and psoas abscess	Thoracotomy for empyema and drainage of iliopsoas abscess plus ampicillin for 3 months	Recovery from his symptoms and tomographic findings
2021—*Streptococcus intermedius* Pleuropulmonary Disease: A Not So Commonly Seen Radiological Picture	Case report	54 years old	Male	Smoking	Right pneumonia with multiple intraparenchymal lung abscesses, empyema, and right pleural effusion	Azithromycin and ceftriaxone initially plus a right apical chest tube and vancomycin and piperacillin‐tazobactam subsequently due to clinical worsening	He died during hospitalization due to a septic shock
2021—*Streptococcus anginosus* Lung Infection and Empyema: A Case Report and Review of the Literature	Case report	37 years old	Male	Chronic bronchitis, colon polyps, smoking	Pneumonia with empyema	CT‐guided placement of pleural catheter with TPA irrigation with subsequently video‐assisted thoracoscopic surgery with lateral decortication and drainage of empyema with the placement of a chest tube plus ampicillin‐sulbactam for 10 days and oral amoxicillin for 2 days at the discharge	Full recovery from his symptoms and tomographic findings
2021—Lateral thoracic artery aneurysm with a lung abscess and empyema caused by *Streptococcus intermedius*	Case report	66 years old	Male	Arterial hypertension, Influenza infection in the previous month	Lung abscess with pyothorax and a pseudoaneurysm close to the lateral thoracic artery	Therapeutic thoracentesis plus ampicillin‐sulbactam, vancomycin, and transcatheter artery embolism	He died during hospitalization
2021—*Streptococcus intermedius*: Unusual presentation and complication of lung abscess	Case report	54 years old	Male	Without relevant medical history	Pneumonia, with lung abscess on the posterior medial aspect of the right lower lobe with discitis from T5 to T6 vertebral bodies	Thoracotomy with pulmonary decortication along with excision of the mediastinal mass plus metronidazole and ceftriaxone during hospitalization and amoxicillin‐clavulanate for 3 weeks after discharge	Recovery from his symptoms and tomographic findings

Abbreviations: CT, computed tomography; MRI, magnetic resonance imaging.

Also, it has been described with Haemophilus parainfluenzae as the causative pathogen in a pulmonary abscess in a 75‐year‐old man.^14^ Besides, other rare infections in body sites due to *S. intermedius* have been described, such as a recently described case in which discitis was reported due to contiguous infection due to a lung abscess of the posterior right lower lobe due to *S. intermedius*.^15^ It has also been described as a causative pathogen in Lemierre syndrome in a 21‐year‐old man with *S. intermedius* bacteremia^16^ and in a 29‐year‐old woman who developed multiple lung abscesses secondary to a uterine empyema caused by an intrauterine device with the S. milleri group as the causative agent isolated.^17^ Furthermore, isolated pulmonary nodules and infective endocarditis due to *S. intermedius* have also been described.^4^, ^18^, ^19^ In our case, blood cultures were performed with a negative result, and no signs or symptoms related to probable or confirmed endocarditis were found. Moreover, a left thoracic artery pseudoaneurysm has been described as a secondary complication of a lung abscess in a 66‐year‐old man.^20^

In regard to SARS‐CoV‐2 coinfection with *Streptococcus intermedius*, we reviewed the available medical literature in the major clinical databases (PubMed, Google Scholar, and SciELO). We found five articles, consisting of three case reports,^21^, ^22^, ^23^ a case series,^24^ and one cross‐sectional study.^25^

The first case report describes a 14‐year‐old African American with SARS‐CoV‐2 infection who developed a severe rapidly progressive complicated sinusitis due to *S. intermedius*.^21^ In this case, the infection progressed to orbital, subgaleal, and intracranial abscesses, requiring surgical intervention and a 4‐week course of intravenous antibiotic therapy, with a resolution of the infection and no neurologic sequelae.^21^ The second case describes the case of an adult older than 65 years with a medical history of SARS CoV‐2 infection who developed a subdural empyema due to SAG, for which he underwent two craniectomies, achieving the eradication of the empyema and clinical improvement, for which he underwent two craniectomies, achieving the eradication of the empyema and clinical improvement.^22^ Finally, the third case describes a 12‐year‐old adolescent with SARS‐CoV‐2 infection who developed appendicitis with perforated gangrenous tissue, from which the purulent material was positive for E. coli, SAG, and SARS‐CoV‐2.^23^

Regarding the case series, this describes the course of six patients with complicated acute sinusitis and SARS CoV‐2 coinfection, of whom SAG was isolated in three patients.^24^ Finally, one cross‐sectional study describes the incidence of beta‐hemolytic streptococci in patients with COVID‐19, finding an isolation rate of only 4.4% for SAG.^25^ In sum, these observational studies show that coinfection between SARS CoV‐2 and SAG has been described, but they do not allow us to affirm that there is an association between SARS CoV‐2 coinfection and SAG.

In relation to SARS‐CoV‐2 infection in pregnancy, it could be higher than in the general population.^26^ However, the risk factors for severe infection (asthma, hypertension, diabetes, overweight, obesity, and being a member of a black or ethnic minority ethnic group) are similar to those in the general population,^26^ however, in our case, none of these risk factors were present. Although vertical transmission is possible, severe neonatal disease seems to be rare, which is consistent with the outcome obtained with the newborn in our case.^26^ Furthermore, the use of corticosteroids anterograde in the case of preterm delivery as well as the treatment of severe COVID‐19 infection appears to be safe for the mother.^26^ On the other hand, it is important to highlight that asymptomatic infections by COVID‐19 in pregnancy seem to be common, but their clinical significance seems to be uncertain.^26^

With respect to the route of infection, the airway was the main route of dissemination for the infection in our case, and despite the fact that certain risk factors such as smoking, alcoholism, dental diseases, chronic obstructive pulmonary disease, malignant neoplasms, liver cirrhosis, and diabetes have been described,^1^ in our case, none of these risk factors were present.

Regarding lung abscess, it is defined as a circumscribed area of pus or necrotic deposits in the lung tissue with the formation of cavities containing necrotic deposits or fluid caused by a microbial infection, and if suspected, chest computed tomography (CT) should be done because CT provides a more accurate anatomical definition than a chest X‐ray and can identify other abscesses and chest lesions that are not clearly delineated on a chest X‐ray.^27^ On CT, an acute lung abscess is usually surrounded by a less well‐defined area of lung parenchyma filled with thick necrotic debris, often involving the apical segment of the inferior lobe of the right lung, which corresponds to the location in our case.^27^ Furthermore, in some cases, CT can distinguish between lung carcinomas and abscesses because malignant lesions have a thicker wall and are more irregular than abscesses.^27^ In our case, this differential diagnosis was considered due to the presence of irregular borders on CT, absence of fever, purulent sputum, and leukocytosis, as well as the persistence of the lesion. However, histological studies ruled out malignancy and confirmed a bacterial lung abscess due to *S. intermedius*.

Concerning the therapeutic approach, the first lines of treatment for susceptible *S. intermedius* strains to beta‐lactams could be penicilins, cephalosporins, and carbapenems.^28^ Besides, a drainage procedure must be considered and performed in most abscess cases.^28^

## 4 CONCLUSION

Pulmonary abscess due to *S. intermedius* is an uncommon clinical scenario, even more so in pregnancy. A few cases have been reported of *S. intermedius* associated with brain and lung abscesses, pleuropulmonary disease, and infective endocarditis. Thus, physicians should take this anaerobic gram‐positive organism into account in these clinical scenarios in order to allow a proper diagnosis and management.

## AUTHOR CONTRIBUTIONS

**Cristian Orlando Porras Bueno:** Conceptualization; data curation; formal analysis; writing – original draft; writing – review and editing. **Sharon Julieth González Trillos:** Data curation; formal analysis; writing – review and editing. **Diana Jimena Cano Rosales:** Conceptualization; formal analysis; writing – review and editing. **Edgar Augusto Bernal García:** Conceptualization; formal analysis.

## FUNDING INFORMATION

None.

## CONFLICT OF INTEREST

None.

## CONSENT

Written informed consent was obtained from the patient to publish this report in accordance with the journal's patient consent policy.

## Data Availability

The data that support the findings of this study are available on request from the corresponding author.
